# Fine-Grained Food Image Recognition: A Study on Optimising Convolutional Neural Networks for Improved Performance

**DOI:** 10.3390/jimaging10060126

**Published:** 2024-05-22

**Authors:** Liam Boyd, Nonso Nnamoko, Ricardo Lopes

**Affiliations:** 1 Apadmi Ltd., Anchorage 2 Salford, The Quays, Manchester M50 3XE, UK; 2 Department of Computer Science, Edge Hill University, St Helens Road, Ormskirk L39 4QP, UK

**Keywords:** food waste management, Image processing, deep learning, recipe suggestion, image recognition, food waste classification

## Abstract

Addressing the pressing issue of food waste is vital for environmental sustainability and resource conservation. While computer vision has been widely used in food waste reduction research, existing food image datasets are typically aggregated into broad categories (e.g., fruits, meat, dairy, etc.) rather than the fine-grained singular food items required for this research. The aim of this study is to develop a model capable of identifying individual food items to be integrated into a mobile application that allows users to photograph their food items, identify them, and offer suggestions for recipes. This research bridges the gap in available datasets and contributes to a more fine-grained approach to utilising existing technology for food waste reduction, emphasising both environmental and research significance. This study evaluates various (*n* = 7) convolutional neural network architectures for multi-class food image classification, emphasising the nuanced impact of parameter tuning to identify the most effective configurations. The experiments were conducted with a custom dataset comprising 41,949 food images categorised into 20 food item classes. Performance evaluation was based on accuracy and loss. DenseNet architecture emerged as the top-performing out of the seven examined, establishing a **baseline** performance (training accuracy = 0.74, training loss = 1.25, validation accuracy = 0.68, and validation loss = 2.89) on a predetermined set of parameters, including the RMSProp optimiser, ReLU activation function, ‘0.5’ dropout rate, and a 160×160 image size. Subsequent parameter tuning involved a comprehensive exploration, considering six optimisers, four image sizes, two dropout rates, and five activation functions. The results show the superior generalisation capabilities of the **optimised** DenseNet, showcasing performance improvements over the established **baseline** across key metrics. Specifically, the optimised model demonstrated a training accuracy of 0.99, a training loss of 0.01, a validation accuracy of 0.79, and a validation loss of 0.92, highlighting its improved performance compared to the **baseline** configuration. The **optimal** DenseNet has been integrated into a mobile application called FridgeSnap, designed to recognise food items and suggest possible recipes to users, thus contributing to the broader mission of minimising food waste.

## 1. Introduction

The global concern of food waste is highlighted in the Food Waste Index Report [1] by the United Nations Environment Programme, revealing a consistent global average of 74 kg per capita of wasted food annually, irrespective of income levels from lower-middle to high-income countries. The volume of food wasted at the household level is much greater when compared to other food supply chain levels [2]. Notably, food loss at the initial stages of the supply chain (i.e., during production, processing, or transportation) is predominant in developing nations [3]. However, this research focuses on the later stages of the supply chain, where food surplus and wastage are primarily observed, and this phenomenon is more prevalent in developed countries [3,4].

There is a general consensus that consumer behaviour is a significant contributor to food waste in developed countries [5]. For example, the National Resources Defense Council [6] reported that a significant amount (30–40%) of food waste in America occurs at the consumer level, with many sources citing “unnecessarily large orders” [7,8] and poor “consumer choices” [9] as major contributing factors. Consumers in the European Union (EU) waste 47 million tonnes of food annually [10]. In research conducted by LEI (Landbouw-Economisch Institut) for the EU Commission [11], Rutten et al. found that 31% of food waste across the supply chain occurs in households. This was corroborated by Becarova et al. [12], who studied consumption behaviour between Dutch and Slovaks; Vanham et al. [13], who quantified EU consumer food waste; and Jörissen et al. [14], who analysed survey results from two EU research centres (in Italy and Germany). The United Kingdom (UK), where this study was conducted, is no exception. Recent reports show that food waste in the UK has increased from 7.2 million tonnes in 2014 [15] to 9.5 million tonnes in 2022 [16]. A study conducted in Scotland alone [17] found that 51% of the food wasted in Scottish households is avoidable, with a large portion stemming from food not being used within its use-by time.

As the global increase in waste is projected to surpass population growth by 2050 [18], food waste is likely to pose significant threats to ecological balance, which will threaten global sustainable development and human well-being [19]. Recognising the impending threat of food waste to ecological balance, various computational solutions have been developed, with advances in computer vision emerging as popular approaches, especially through deep learning methods. However, most of this research on food waste reduction has frequently neglected the pivotal role of consumer behaviour (the driving force behind this research), often concentrating on the management of discarded food. Specifically, many approaches applying deep learning to separate household food waste images into recyclable and non-recyclable items [19,20] have been proposed, effectively replacing manual sorting by individuals and streamlining the waste management process. The research presented in this study suggests that deep learning applications on food images also hold promise for innovative solutions, which encourage consumers to optimise the consumption of available food items. For example, a mobile application called ‘emptymyfridge’ (https://www.emptymyfridge.com/ (accessed on 29 February 2024)) employs image processing through bar code scanning to prioritise recipes that match particular food items. This object recognition aspect enhances user convenience but unfortunately, the application was developed on proprietary datasets, and the underlying methods are not openly accessible to the research community. In fact, this is a common issue in consumption-stage food waste reduction interventions as recent reviews show that existing applications either lack method clarity [21] or provide little or no robust evidence of results [22]. Moreover, existing open-access datasets commonly used for food image recognition tasks (such as ‘Food-101’ [23], ‘Food-5K’ and ‘Food-11’ [24]) are aggregated into food categories (e.g., meat, dairy, fruits, etc.), making them impractical for individual food item recognition experiments. Thus, this study aims to “bridge the gap in available datasets” and contribute a more “fine-grained” and “transparent” approach in utilising existing computer vision technologies for food waste reduction, with emphasis on practicality in a mobile application and the substantial influence in inducing consumer behaviour changes.

In this study, a method based on a convolutional neural network (CNN) is presented that combines computer vision technology with behaviour-orientation strategies to facilitate food waste reduction. For the computer vision aspect, the performances of various CNN architectures are evaluated on a multi-class image classification task performed on a custom fine-grained food dataset [25]. A custom dataset consisting of 20 food item classes was developed specifically for this study due to the limitations of existing food image datasets [26,27] that were primarily categorised into food groups (e.g., dairy, meat, fruit, etc.) rather than individual food items. The evaluation was based on accuracy and loss metrics. To achieve the behaviour-orientation aspect of this study, the top-performing CNN model has been integrated into a mobile application called FridgeSnap [28], which was designed specifically to identify food images captured on user device cameras and recommend suitable recipes, thus encouraging less wasteful consumer behaviour. To encourage transparency and allow for the reproducibility of the experiments carried out, the demonstrator software, along with the Python script to replicate the method, has been published for the research community on GitHub [29].

This study acknowledges that the idea of image classification has been carried out, to a large extent, within the area of food and dietary circumstances. However, this study makes two new contributions as follows:

1. Fine-grained food image dataset: A custom multi-class dataset is developed that consists of 41,949 images belonging to 20 different individual food item classes [25]. The existing multi-class food image datasets for classification tasks are categorised by food groups such as dairy, fruits etc. The experiments carried out utilise fine-grained food images of singular food item classes.
2. Transparent benchmark method: The experimental method, along with code to replicate the experiments, has been published [28], providing a benchmark to objectively gauge the progress of new methods for fine-grained multi-class food image classification. Underlying methods in existing consumption-level applications for food waste reduction lack clarity due to being concealed in proprietary software, which makes it challenging to make a meaningful comparison of approaches and results.

The rest of the research is structured as follows: Section 2 provides details about related work and the necessary background for the techniques and tools used in this experiment. The experimental data and methodology approach, including details about the experiment setup and evaluation measures, are presented in Section 3. Findings and results, including potential threats to validity, are discussed in Section 4. Section 5 presents the findings in the context of the food waste domain while Section 6 summarises the study and points out future work.

## 2. Background and Related Research

For many, the issue of food waste is ignored, which is the key motivation for this study. Food wastage has occurred at both the consumer and producer levels. At the consumer level, in the year 2010 alone, ‘7.2 million tonnes’ of both food and drink waste was generated within the UK [30]. This issue goes further than damaging the economic status of the UK, with an annual average of GBP 730 per family being wasted, based on statistics from WRAP [31]. This has environmental implications, as statistics indicate the production of approximately 17 million tonnes of CO_2_ in greenhouse gas emissions [30]. While these statistics are derived from data related to 2010 and 2011, it is reasonable to assume that these figures have risen with the population growth. Furthermore, increasing food prices could have a resulting impact on the growth of food waste because ‘offers’ or ‘deals’ on items may result in the consumer purchasing more of the item than needed. Food waste is not just caused by over-purchasing, with Visschers et al. [32] highlighting that families that have children have been found to waste more food than families without children. This is due to children potentially being indecisive with regard to eating certain items of food, or the amount of food they will eat, creating a further generator of food waste. Although international methods have been introduced within the past 13 years to reduce food waste, these have proved somewhat ineffective with the issue continuing to grow and the impact it has on both the economy and environment further increasing. Allison et al. [33] highlighted recent figures to support this, showing that ‘9.5 million tonnes’ of food waste is generated, increasing from ‘7.2 million’ in 2010. Due to this, a greater call is required for innovative ways and methods to be introduced that can tackle food waste.

As the issue of food waste has continued to increase, the amount of initiatives introduced globally has equally increased. Some of these initiatives have remained at the framework level without practical implementation, while others have been fully realised through computational solutions. For example, Principato et al. [34] conducted a study that focused on understanding the reasoning behind why food is wasted, focusing on young individuals in Italy. The study found that a large contributor to the issue was due to a lack of knowledge, or being misinformed regarding a certain topic. Individuals understand that food must be discarded if it may no longer be fresh or if the item passes a certain expiration date. Although both reasons could be seen as valid arguments for food to be wasted, the authors argue in favour of a framework that provides additional information and guidance on how to make food last longer. This was corroborated by Poyatos-Racionero et al. [35] who proposed a framework for intelligent packaging where information regarding the safety and edibility of an item is presented on the packaging. This includes numerous types of information such as time, temperature, and freshness indicators. The authors argued that consumers will gain a greater understanding of when an item must be discarded if these indicators are available on the packaging at the time of purchase. Other frameworks have been proposed since then, including Lim et al. [36] who developed a ‘social recipes’ framework aimed at reducing food waste through recipe sharing. The core idea is that recipes can be shared through a community-based environment to provide users with tips about what they can do with leftover food items. This idea of ‘social recipes’ is similar to the method presented in this study but remains at the framework level without practical implementation.

Many initiatives have been fully realised through computational solutions. For example, ‘SuperCook’ (https://www.supercook.com/ (accessed on 29 February 2024)), an application that uses manual text or voice submission of ingredients, can then generate recipes. These recipes are categorised into areas such as “Breakfast and brunch”. ‘emptymyfridge’ is another application that uses manual ingredient submission to generate recipes that can be saved. This application uses a form of image processing to allow for barcodes of items to be scanned and uploaded. ‘Plant Jammer’ (https://www.plantjammer.com/ (accessed on 29 February 2024)) uses a similar method of manually inputting ingredients by selecting ingredients on a landing page, these are then turned into recipes, which can be filtered based on difficulty or dish type. The recipes generated by ’Plant Jammer’ are generated inside the application and do not require the use of an external browser or application. Despite the promising claims reported about these tools, their potential in terms of performance is often achieved on proprietary datasets and the underlying methods remain unavailable to the research community. In fact, most of these tools focus more on recipe suggestions based on manually entered food items, which has many limitations including the database dictionary size. There is no usage of image-processing-related technologies within any of the aforementioned applications. As a result, it has not been possible to make a sound comparison of different tools that promote consumption-stage food waste reduction.

That said, several studies have been published to address the issue of food waste databases, including benchmark results of popular methods to provide a standard experimental platform for assessing progress. Image processing has been, to a greater extent, the popular approach within the area of food waste management and/or classification. The development of image classification models relies heavily on the implementation of a categorised training dataset, which includes images of the item to be classified with appropriate label(s) to identify them. Several research studies in this area have used pre-developed benchmark datasets such as ‘Food-101 [23], ‘Food-5K’, and ‘Food-11’ [24]. Food-5K contains images of both ‘food’ and ‘non-food’ item classes with 1000 food items with an equal split of 50% between food item classes. Food-11 contains 16,643 food images with 11 categories: *Bread, dairy products, desserts, eggs, fried food, meat, noodles-pasta, rice, seafood, soup, and vegetable-fruits*. Food-101 contains 101 food categories, with a dataset size of 101,000 images. Each of the food categories contains 750 training images and 250 testing images. The Food-101 dataset contains pre-made food item categories, such as *spring rolls, pizza, and spaghetti bolognese*. Studies that utilised benchmark datasets include one by Şengür et al. [37], who used the Food-5K, Food-11, and Food-101 datasets to carry out food image classification. The papers achieved a result of 99.20% using Food-5K, 89.33% when using Food-11, and 79.86% using Food-101; Hooker et al. [38] used the Food-101 dataset and ResNet-50 to achieve a result of 84.54%. Many other research studies, including Aguilar et al. [39], Tan and Le [40], and Dwibedi et al. [41], contributed to the field. Results from these studies have achieved accuracy values between 76.7% and 97.92%. While the benchmark datasets, accompanied by evaluation methodologies and performance studies, provide multiple benchmarks to objectively assess the progress of food waste initiatives, the class composition of these datasets (i.e., food item groups rather than singular food item classes) does not support fine-grained, consumption-level experiments that could influence behaviour change toward reducing food waste.

It is important to note that other similar datasets have been developed to address specific but related challenges. For example, Chun et al. [42] developed a custom dataset based on Korean food images for classification purposes. This dataset, generated using a web scraper, consists of 150,610 food images. Each image is categorized into one of 150 traditional Korean food classes, including *Kimchi, Bap, Twigim, and Jeongol*. The resulting classification model trained on this dataset achieved an accuracy of 81%.  Mezgec and Seljak [43] also developed a custom dataset of 631 images using a script that searches the internet to generate the dataset. This was utilised to perform the ‘beverage’ vs. ‘food’ image recognition task, resulting in a model with 92.18% accuracy. In fact, many studies found in the literature have combined benchmark dataset(s) with custom-generated ones.

 Kagaya and Aizawa [44] used a combination of three datasets (Food-101, Caltech-256 [45], and a custom dataset collected from Instagram) to perform binary classification tasks. Caltech-256 is used as the non-food dataset alternative to Food-101, which contains images of just food items. Moreover, 28,322 non-food images are extracted and used from the Caltech-256 dataset. The Instagram dataset contains 4230 food images and 5428 non-food images. The food images collected are of full meals or pre-made food, such as ice cream or cake. The CNN model trained on the data achieved 99.1% accuracy.

Islam et al. [24] compared the performances of several CNN models on three different datasets. The idea was to investigate performance variation between binary vs. multi-class classification tasks. The best accuracy result on binary classification is 98.37% with a runtime of 12,509 s, while the multi-class task involving 11 food classes achieved a decreased accuracy of 83.52% with slightly more runtime (125,134 s). Interestingly, the results of the multi-class tasks showed a continuous decrease in accuracy as the number of classes increased. Specifically, with 22 classes, the accuracy dropped to 58.72% accompanied by a significantly increased runtime of 423,316 seconds. This research highlights the issues of decreased accuracy and increased runtime when working with a larger number of classes. However, this issue is not replicated in the multi-class study by Chaitanya et al. [23], which involved 20 and 25 food item classes extracted from the Food-101 dataset. The authors explored several pre-trained CNN architectures on the data, with the most accurate model achieving an accuracy of 92.23% for 20 classes. The accuracy was reduced to 91.46% when using 25 food classes. This underscores the need for an exploratory research approach when using pre-trained CNN architectures to determine the most suited model for the dataset.

Across the literature, a number of pre-trained CNN architectures have been explored for performance on various datasets, including AlexNet [46], InceptionNet [47], NutriNet [43], GoogLeNet [48], ResNet [49], etc. These architectures, renowned for their success in image-related tasks, have not only excelled in food image recognition but have also catalysed the development of bespoke models in related studies. For example, their proven success in image-related tasks has paved the way for the creation of a specialised application for domestic waste management [19] where a bespoke CNN trained on a domestic waste dataset, including food images, was used to separate waste into recyclable and non-recyclable. In fact, the success of specialised CNNs for image-related tasks extends beyond food waste domains as shown in [50], where they have proven instrumental in education for recognising students’ emotions in online classrooms. This versatility shows the adaptability of pre-trained CNNs, and their potential to transcend specific domains and provide valuable insights across a spectrum of applications.

For brevity, the review presented in this study focuses only on pre-trained CNN applications in the culinary domain. InceptionNet was used the most in research by Chun et al. [42] and Chaitanya et al. [23], with results of 81% and 97%, respectively. Other literature discusses the use of various CNN architectures. For example, NutriNet achieved an accuracy of 92.18% [43]; AlexNet, GoogLeNet, and ResNet-50 reported an average accuracy of 58.72% [24], a ‘Bag of Texons’ approach yielded an accuracy of 87.44% [51], and the ‘Network in Network’ approach achieved the highest accuracy of 99.1% [44].

Results from the literature indicate that an increased number of classes may decrease accuracy, as evidenced by two different studies that investigated performance across various classes [23,42]. This presents the idea that the accuracy of a model not only depends on the pre-trained CNN architecture but also on the data size, the number of classes, and their distribution in the dataset. In fact, the differences in accuracy observed in the various studies highlight the importance of an exploratory approach taken in the research presented in this study. As the focus of this research was to perform classification on singular food items, a fine-grained dataset was deemed necessary for the experiments. This is because popular benchmark datasets such as ‘Food-101’ [26] and ‘Food-11’ [27] feature large groups of food, focusing on full plates rather than singular food items.

## 3. Methods and Materials

This section presents the methods and materials including details of the developed fine-grained dataset, the data preprocessing steps undertaken to set up the experiments, and the research method adopted to address the study aims.

### 3.1. Research Methodology

When addressing the aim of bridging the gap in available datasets and training a CNN model capable of automatically identifying food items from images, a comprehensive design science approach was employed. The initial phase involved the meticulous creation of a diverse dataset of food images to provide fine-grained labels for individual food items (see Section 3.2). This dataset served as the foundation for training the CNN model.

The subsequent steps of the design process focused on the exploration of seven existing CNN architectures, including VGG versions 16 and 19 [52], InceptionNet [47], ResNet [49], MobileNet [53], DenseNet [54], and Xception [55]. An exploratory phase was crucial to understanding the performance nuances of these architectures in the specific context of fine-grained food item recognition, which involves image classification. Through hyperparameter tuning, the selected architectures were optimised for the classification task. This iterative approach allowed for a systematic evaluation of each model’s performance, considering three metrics (i.e., accuracy, loss and experiment runtime).

The design process was inherently iterative, featuring a feedback loop that incorporated insights gained from evaluations to refine the model continuously as shown in Section 3.3. This iterative nature ensured that the CNN model was continually improved for enhanced accuracy and efficiency. The final selection of the most optimal model took into account a balance between performance metrics and computational efficiency, aligning with the practical requirements of integration into a mobile application. Explicit integration of the CNN model into a mobile application called FridgeSnap has been published as standalone software [28], including details of the dataset [25] and the source code [29], to encourage reproducibility. However, a brief discussion is provided on the application in this study to mark a seamless transition from the development phase to practical implementation. Users can leverage the application by capturing photos of their food items, initiating the trained model through a button to identify the items, and receiving tailored recipe suggestions. This integration not only represents the successful completion of the design process but also emphasises the practical utility of the CNN model within a real-world application.

### 3.2. Dataset

The experimental dataset collection commenced with systematic retrieval of images via the Google Images application programming interface (API), which enables users to scrape images from a Google Images search results page. Initially, search terms related to 20 food items were entered into Google Images. These terms included expressions like ‘[food item] in the fridge’ or ‘[food item] isolated on white background’, where food item represents one of the 20 food items considered. While the initial attempt involved the Google Images API, it was later found that this approach did not yield satisfactory results, either due to lower image quality or an insufficient number of images for building a comprehensive dataset. Subsequently, a more effective manual scraping technique was employed. Food items, such as ‘banana in fridge’ or ‘banana isolated’, were manually searched, and images were saved by right-clicking and downloading. This method provided better control and resulted in a more extensive collection of high-quality images for the developed dataset. Given the varying availability of each food item, a limit of 2500 images per food item was imposed to prevent undue class imbalance. It is noteworthy that not all classes reached this predetermined limit due to discrepancies in availability. Subsequently, to ensure dataset integrity and minimise redundancy, a meticulous removal of duplicates was executed. By leveraging both matching file names and the ImageHash Python library, which offers support for a spectrum of image hashing algorithms, including average, perceptual, difference, wavelet, HSV-colour, and crop-resistant methods, duplicates were efficiently identified and eliminated. This rigorous curation process resulted in an overall reduction across all classes, culminating in a curated fine-grained dataset comprising 41,949 unique images of 20 food classes. The retrieved images were then downloaded, converted to .jpg format, and resized during experiments into dimensions of 80×80, 120×120, 160×160, or 224×224.

**Figure 1:**
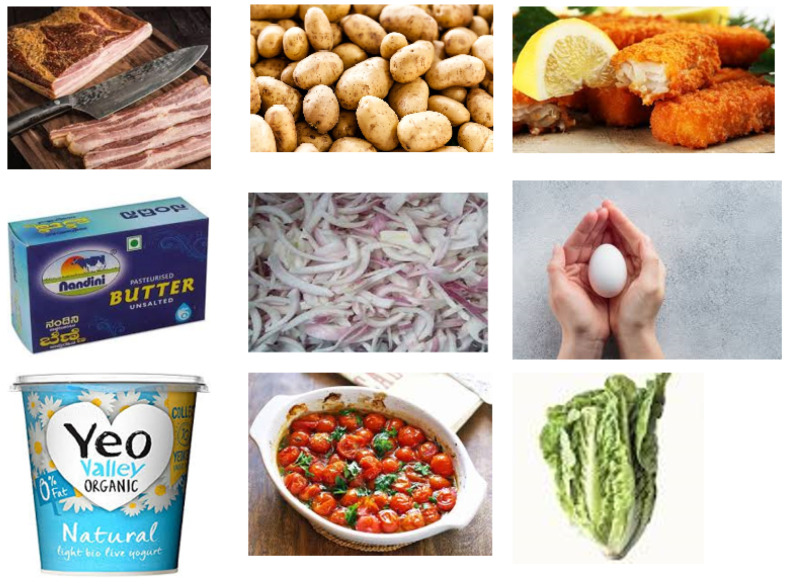
Sample of images contained within the dataset.

The retrieved images included as many variations of each food item as possible including images of both processed and unprocessed versions, varying camera angles, juxtapositions with other objects, etc. Example images from the dataset are shown in Figure 1.

However, it is important to acknowledge the inherent limitations, recognising that certain nuances, such as fuzziness and background illumination, may not have been comprehensively accounted for. These nuances can be easily obtained through image augmentation [56], a useful technique used in image processing to increase the diversity of the training dataset through simple transformations such as geometric and colour space changes, image cropping, noise injection, and random erasing. This technique has been included as a future research direction.

The amount of data contained within each class, including the split for experiments, is presented in Table 1.

The multi-class food image has been made publicly available on the Kaggle data repository [25] to facilitate the reuse and reproducibility of the experiments carried out.

For experimental purposes, the data were split into training (80%) and validation (20%). This is a well-established method in classification tasks, offering a balanced approach to model development and assessment. To further ensure the robustness of experiments, within this study, a testing subset is employed within the training data. This subset, comprising 20% of the training set, serves as an intermediary checkpoint for model evaluation during the training process. It helps prevent overfitting, which is a common pitfall where a model becomes too tailored to the training data and struggles to generalise to new instances.

Importantly, the use of a validation subset strategy addresses the classic issue of ‘test set validation’, which is a practice that inadvertently biases model evaluation because the test set has been compromised during training. Validating models on the test set can lead to an inflated sense of performance, as models are indirectly exposed to the test data during training. By holding out a validation subset within the training data, it is ensured that the test set remains entirely unseen until the model’s training is complete. To maintain a representative sample distribution, the data split was stratified across the 20 classes. This means that each class is proportionally represented in both the training and test sets, avoiding potential biases that could arise from an uneven distribution.

### 3.3. Experiments

**Figure 2:**
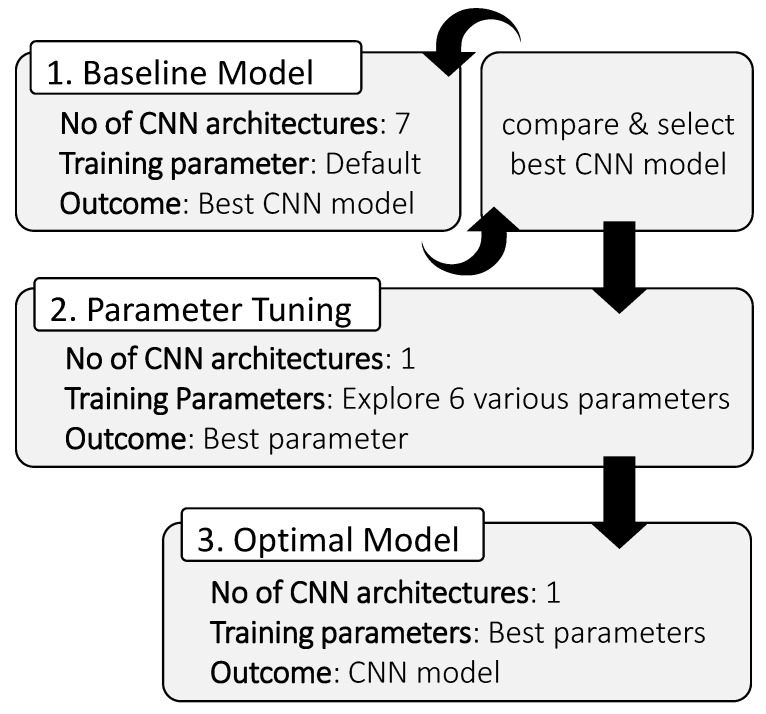
High-level diagram of method steps.

The experiments described in this section are grounded on the underlying hypothesis that multiple factors contribute to the optimal performance of CNN models as observed in the literature. Thus, multiple classification tasks involving seven standard CNN pre-trained models were performed on the dataset presented in Section 3.2 to identify the most optimal setting for the classification task. This **optimal** model was derived through a step-wise development approach as illustrated in Figure 2.

All three steps involved classification tasks with a single pre-trained CNN architecture, except step one, where seven CNNs were explored namely, VGG versions 16 and 19, InceptionNet, ResNet, MobileNet, DenseNet, and Xception. The overarching purpose was to compare and select the CNN model(s) with optimum performance on the test dataset. However, the choice of CNN architectures was deliberate, driven by various considerations, most notably their high flexibility in accommodating diverse types of image data and tasks. Flexibility is a crucial criterion in this study, considering the diverse nature of food images, encompassing different shapes, sizes, and colour distributions. These networks have demonstrated adaptability across a wide range of image classification tasks, making them suitable candidates for handling the intricacies present in food classification. Furthermore, the selected CNN architectures, particularly VGG, ResNet, and InceptionNet, have established themselves as benchmarks in the field of deep learning. Their widespread adoption and extensive utilisation in various image classification competitions and research endeavours underscore their reliability and strong performance across diverse datasets. Importantly, these architectures have achieved such versatility through pre-training on massive datasets like ImageNet, which comprises millions of images, including a vast array of food items.

The performance of these CNNs on multi-class classification tasks has been well-documented in previous studies, as shown in Section 2. For instance, ResNet, known for its deep residual learning and effectiveness in addressing the vanishing gradient problem, along with DenseNet, recognised for its dense connectivity pattern, are particularly suitable for tackling intricate multi-class classification tasks. Similarly, InceptionNet’s inception modules and MobileNet’s lightweight design have shown promise in accurately classifying images from a broad spectrum of categories. In fact, each of the seven CNN architectures offers distinct approaches to deep learning.

DenseNet [54], for instance, introduces dense connectivity, ensuring each layer is directly connected to every other layer, promoting feature reuse and efficient gradient flow. Conversely, Xception [55] extends the Inception architecture [47] by utilising depth-wise separable convolutions, enhancing computational efficiency without compromising performance. VGG16 and VGG19, developed by the Visual Geometry Group at Oxford University [52], follow a simpler design characterised by cascading convolutional and max-pooling layers, followed by fully connected layers. VGG19 boasts additional layers compared to VGG16, resulting in increased model complexity. InceptionNet [47], also known as GoogLeNet, revolutionised CNN architectures with its inception module, enabling convolutions at multiple spatial scales within the same layer. It leverages 1 × 1 convolutions for dimensionality reduction, reducing computational complexity. MobileNet [53] is tailored for mobile and embedded devices, emphasising low latency and computational cost. Its design incorporates depth-wise separable convolutions to minimise parameters and computational overhead. ResNet [49] introduces residual connections, allowing information to bypass certain layers, mitigating the vanishing gradient problem and enabling the training of extremely deep networks. Each architecture offers unique features and optimisations to address specific challenges in image classification and recognition tasks.

Another crucial factor that influenced this study is the availability of pre-trained weights for these networks on large-scale image datasets like ImageNet. This pre-training helps in leveraging transfer learning, allowing the networks to extract relevant features from the developed food dataset despite its smaller size. This approach is especially beneficial when working with limited data, as is often the case in specialised classification tasks.

It is important to note that the classification process in each development step (steps 1 to 3) in Figure 2 was conducted on the experimental data split shown in Table 1. The underlying CNN is trained on the training dataset with the validation set used to explore performance over several pre-set epochs. Then, the best model is evaluated on the holdout testing dataset.

#### 3.3.1. ^*STEP* 1^Baseline Model

As the study was conducted on a custom fine-grained dataset, there is no existing benchmark study to compare against the **optimal** model (described in Section 3.3.3). Thus, this step began with a comparative experiment to evaluate the performances of the seven CNN architectures on the experimental dataset, ultimately leading to a **baseline** model. It is important to note that a fixed experimental setting was used in this step for each CNN, including ‘160×160’ image size, batch size = ‘16’, epoch = ‘50’, and dropout = ‘0.5’. The learning rate is set to ‘0.001’ to minimise the model’s weight updates throughout the training process. When using a learning rate of 0.001, the weights are updated by 0.1% (0.001) of the gradient of the loss function with respect to those weights during each iteration of training. This means that the weight updates are relatively small compared to higher learning rates. Since the weights are adjusted by a small amount, the model does not learn much from the training data and, thus, will not adapt to intricate patterns or improve its performance on the task by much. As a starting point, the root mean square propagation (‘RMSProp’) [57] optimiser and rectified linear unit (ReLU) [58] activation function were used. The RMSProp was chosen arbitrarily and ReLU has become widely adopted as the default activation function for training deep neural networks due to its versatility across various task domains and network types, as well as its low computational complexity. The model that achieved the best performance at this step was used in subsequent steps to derive the **optimal** model. Results of the **baseline** experiments are detailed in Section 4.1, which shows that the DenseNet [54] model achieved the best performance at this step. Thus, subsequent experiments utilised the DenseNet architecture.

#### 3.3.2. ^*STEP* 2^Parameter Tuning

In this step, the best model (from the previous step) was subjected to hyperparameter tuning, which involves multiple iterations of experiments where different combinations of parameters are explored to find the best configuration that maximises the model’s performance. This aim is to strike a balance between underfitting (too simple) and overfitting (too complex) by finding the optimal values for the parameters. The optimisation experiments were exploratory and conducted iteratively using a semi-automated approach as follows:

 First, seven optimisation algorithms on the CNN were explored including stochastic gradient descent (‘SGD’) [59], ‘RMSProp’, ‘Adagrad’ [60], ‘Nadam’ [61], ‘Adam’ and ‘Adamax’ [62]. These algorithms adjust the weights and biases of the network during training in order to minimise the loss function and improve the model’s predictive accuracy.
 The best optimiser was used subsequently to test the CNN on four image sizes including ‘80×80’, ‘120×120’, ‘160×160’ and ‘224×224’. This allows for the evaluation of the model’s robustness and generalisation across varying resolutions, which is important for assessing performance on different devices and real-world scenarios. This is particularly important because the model was intended for use within FridgeSnap [28], a mobile application that suggests recipe(s) based on food image(s) taken from the user’s device camera.
Finally, this study explored performance with five activation functions including ‘ReLU’, ‘Sigmoid’, ‘Tanh’, ‘Swish’ [63] and Gaussian error linear unit (‘GELU’) [64].

#### 3.3.3. ^*STEP* 3^Optimal Model

In this step, an **optimal** model was implemented based on the best parameters identified in the previous step (see Section 4.3). The resulting model was evaluated and compared to **baseline** performance according to the metrics defined in Section 3.4.

### 3.4. Evaluation Metrics

**Figure 3:**
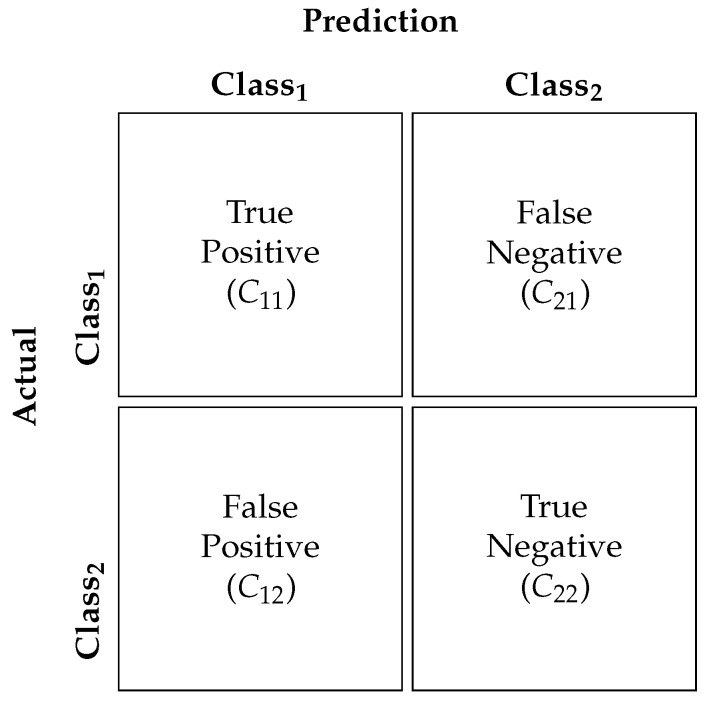
Simple 2×2 confusion matrix.

The evaluation of CNN classification models commonly revolves around aggregate metrics derived from a confusion matrix shown in Figure 3.

$C_{11}$ represents the number of Class_1_ predicted correctly; $C_{22}$ is the number of Class_2_ predicted correctly; $C_{12}$ is the number of Class_1_ predicted incorrectly; and $C_{21}$ is the number of Class_2_ predicted incorrectly.

In this study, the ’ accuracy’ metric was defined as a calculation of how often the predicted class label matches the actual class label, reflecting the overall correctness of a classification model. Its value deduced from the confusion matrix can be represented mathematically as Equation (1) [65]:

(1) $$\mathrm{Accuracy}=\frac{C_{11}+C_{22}}{C_{11}+C_{12}+C_{21}+C_{22}}$$

Additionally, the loss function, which quantifies the discrepancy between predicted and actual labels was computed. For this, ‘cross-entropy’ loss was used, which is one of the most popular loss functions for classification tasks. It calculates the negative log-likelihood of the predicted class probabilities given the true class labels. This penalises larger deviations between predicted and true probabilities, thus encouraging the model to make confident predictions. Cross-entropy loss is not directly derived from the confusion matrix but rather from information theory and probability theory expressed mathematically in Equation (2) [65]:

(2) $$H\left(p,q\right)=-\sum_{i}p\left(i\right)log\left(q\left(i\right)\right)$$

where H(p,q) is the ‘cross-entropy’ between the true probability distribution *p* and the predicted probability distribution *q*. *i* represents the individual classes. p(i) is the true probability of class *i*. q(i) is the predicted probability of class *i*.

In the context of the CNN classification tasks, q(i) represents the output of the network after applying a softmax activation function, which transforms the network’s raw output scores into a probability distribution. The ‘cross-entropy’ loss penalises larger differences between the predicted probabilities q(i) and the true probabilities p(i), encouraging the model to adjust its parameters to minimise the overall loss.

### 3.5. Experimental Setup

This section presents detailed information about the experiment setup to facilitate the reproducibility of results. The experiment was conducted with Python programming language [66]. VGG versions 16 and 19 [52], InceptionNet [47], ResNet [49], MobileNet [53], DenseNet [54], and Xception [55] were executed using their default TensorFlow implementations [67], which use the keras.applications module to load pre-trained CNN models.

For VGG versions 16 and 19 models, the VGG16 and VGG19 classes, respectively, from the tensorflow.keras.applications module were used. From the same module, this study used the InceptionV3 class for the InceptionNet model; ResNet50 class for the ResNet model; MobileNet class for the MobileNet model; DenseNet121 class for the DenseNet model, and the Xception class for the Xception model.

These classes allow for multiple parameters to be set during initialisation, such as the weights and include_top. The weights parameter is used to specify whether to load the pre-trained weights for the model and it can take the values ‘ImageNet’ to load the weights trained on the ImageNet dataset, or ‘False’ to train without any pre-trained weights. The include_top parameter is used to indicate whether to include the fully connected layers of the model or not. It can take two values—‘True’, which includes the fully connected layers, or ‘False’, which excludes the fully connected layers. This study set the weights and include_top parameters to ‘ImageNet’ and ‘False’, respectively, for all the models.

The ’ImageNet’, parameter allowed for pre-trained weights from the ImageNet dataset to be loaded. However, the training parameters of each of the layers were set to non-trainable by indicating ‘False’ for include_top parameters. This step deactivates the backward propagating step in the CNN models so that only the features based on the model, which was trained on the ImageNet dataset, were extracted for further processing. This is shown on *line 7* of code Listing 1, which represents an example Python code using DenseNet Architecture. The snippet in *line 10* allows for iteration through the model layer using a for loop. *Line 6* snippets were used to set the trainable parameter of each layer to ‘False’. The snippets in *lines 13–17* add additional layers on top of the base model. Specifically, *line 14* creates a new layer by applying the ‘Flatten’ operation to the output of the base_model. The Flatten layer reshapes the output tensor from the bas_model into a one-dimensional tensor (i.e., a vector). This is typically done when transitioning from convolutional layers to fully connected layers in a neural network architecture. In *line 15*, a new fully connected layer (‘Dense’) with 1024 units and a ’swish’ activation function is added. The output of the previous Flatten layer ‘x’ serves as the input to this new fully connected layer. Each unit in this layer is connected to every neuron in the previous layer, and the ’swish’ activation function is applied element-wise to the output of this layer. *Line 16* applies dropout regularisation to the output of the previous fully connected layer ‘x’. Dropout is a technique used to prevent overfitting by randomly setting a fraction (here, 50%) of the input units to zero during training, which helps prevent units from co-adapting too much. Finally, a dense layer with 20 units and a ’softmax’ activation function is added. This layer produces the final predictions of the model. Each unit in this layer represents a class, and the ’softmax’ activation function is applied to convert the raw output into probability scores for each class, ensuring that the output values sum up to 1.

**Listing 1.** Example Python code with DenseNet.

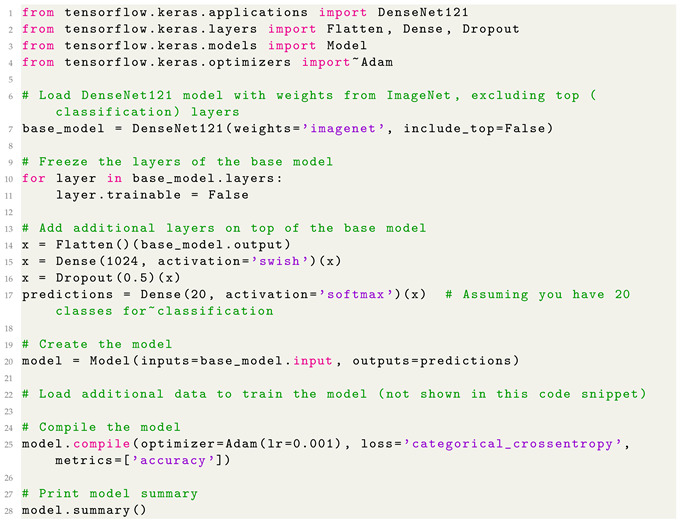



Freezing layers in a pre-trained neural network is a common practice used to prevent them from being updated during the training process. This was specifically important in the experiments because early layers of pre-trained CNN capture broad and low-level image features, which have broad applicability. Freezing these layers allowed for leveraging the pre-trained CNNs’ feature extraction capabilities while focusing training efforts on the specific task, especially given the modest dataset available for experiments. In addition, the training data used may be closely aligned with ImageNet data so freezing the layers helps to avert possible overfitting and curtail trainable parameters, diminishing the risk of the model memorising data. The process also accelerates training since static layers do not undergo weight updates, thus expediting computations.

As explained in Section 3.3.1, the baseline experiments were conducted with batch size: ‘16 ’, epoch: ‘50’, image size: ‘160×160’, epochs: ‘50’, optimiser: ‘RMSProp’, activation function: ‘ReLU’, dropout: ‘0.5’ and learning rate: ‘0.001’. However, the best model from this method step (i.e., DenseNet—see Table 2) was subsequently fine-tuned (in Section 3.3.2) to identify optimal parameters for training and the outcome is presented ‘later’ in Table 3. These parameters (also noted in Section 3.3.3) were used to develop the **optimal** model for comparison with the **baseline**. For the **optimal** model, the batch size was set to ‘32’ and the number of epochs was set to ‘50’. These values were chosen after testing increasing and decreasing the value and observing the difference this had on the accuracy and the runtime of the model. The batch size of ‘32’ was chosen as it generated the most accurate results without having a negative impact on the runtime of the model or the graphics processing unit (GPU) of the training machine which has the following specification-Processor: Intel(R) Core(TM) i7-10750H CPU @ 2.60GHz; GPU: NVIDIA GeForce RTX 2070 with Max-Q Design 16GB; RAM: 16GB. In some cases, it was observed that the model would run into memory issues if the batch size was increased to a larger number.

For the experiments, the dataset was divided into three subsets: training, testing, and validation as shown in Table 1. Initially, 20% was set aside for validation, while the remaining 80% was further partitioned into 80% for training and 20% for testing (during training and optimisation). For each training iteration, the testing set was used to determine the best model (over a pre-defined set of epochs) on which to test. The validation set was kept unseen during training to avoid overfitting. The validation was implemented using the tf.keras.callbacks.EarlyStopping class, which is a callback monitor used to stop training if performance does not improve for a certain number of epochs, thereby preventing overfitting.

This study uses the TensorFlow built-in tf.keras.metrics.Accuracy class to compute the ‘accuracy’ metric while the tf.keras.losses.CategoricalCrossentropy class was used to compute the ‘loss’ metric.

## 4. Results

This section presents the results of experiments to fulfil the method steps described in Section 3. For clarity, the results are presented in separate sections, from Section 4.1 to Section 4.3, each representing outcomes of the experimental steps illustrated in Figure 2. A more in-depth analysis and interpretation of the results in the context of the study’s goal—to assist consumers in reducing food waste through recipe suggestions is provided in Section 5.

### 4.1. Baseline Model_STEP 1_

Table 2 presents the performances of all seven pre-trained CNNs (in descending order of ‘Validation Accuracy’) when applied to the experimental dataset.

The DenseNet model (**baseline**) emerged as the front runner, boasting a training accuracy of 74% and validation accuracy of 68%. This model achieved a lower training loss of 1.25, indicating that it learned efficiently without overfitting. The runtime of 4150.94 ms demonstrates that the model strikes a good equilibrium between performance and computational efficiency.

In contrast, other models like Xception, VGG-16, InceptionNet, MobileNet, VGG-19, and ResNet exhibit varying levels of performance across training and validation. Although some models exhibit higher training accuracy, their validation accuracy is comparatively lower, implying overfitting or a lack of generalisation. These findings stress the importance of the data split method used in this experiment to ensure that models are evaluated appropriately to ensure that they not only achieve high accuracy on training data but also perform well on unseen test data, as demonstrated by DenseNet. The purpose of generating and training the baseline model is to allow for the most accurate CNN to be determined based on a pre-set value of parameters. The highest performing CNN from this test is carried forward and a number of set parameters are then tested to evaluate the performance. It could be argued that this testing phase is not necessary and all CNNs should be tested when carrying out parameter testing. However, as the parameters applied to the CNN testing were basic and not complex, including low batch sizes, number of epochs, and standard image sizes, any low-performing CNNs that were observed may not gain much from increasing the parameters slightly. In the example of ResNet, the accuracy across both training and validation was low with values of ‘0.09’ and ‘0.11’ respectively, with a high runtime of ‘4919.54 s’. Increasing various parameters such as increasing the value of epochs, or the batch size, may not have much of an impact on the ResNet results. As such, carrying out the CNN testing early can determine whether a certain CNN is effective for this type of image classification training and the type of dataset used. Although some earlier observed literature may determine that ResNet performs well, it is dependent on both the dataset and the situation it is applied. As such, this early CNN testing phase allows for those CNNs that do not conform to the situation to be eliminated early, saving both time and resources.

This research considers the best-performing CNN (i.e., DenseNet) as the **baseline** model optimised in subsequent experimental steps and used for measuring the contributions of the **optimal** approach presented in Section 4.3.

### 4.2. Parameter Tuning_STEP 2_

Table 3 summarises the results of parameter tuning on the DenseNet CNN architecture. Various parameters, including the optimiser, image size, dropout, and activation, were explored.

The Adagrad optimiser demonstrated superior performance compared to others. This optimiser excelled in both training and validation metrics, with a training accuracy of ‘0.99’, training loss of ‘0.04’, validation accuracy of ‘0.76’, and validation loss of ‘0.96’.

When considering different image sizes, the model’s performance varied. The  224×224 image size proved to be optimal, yielding a training accuracy of ‘0.99’, training loss of ‘0.01’, validation accuracy of ‘0.78’, and validation loss of ‘0.92’. This suggests that a larger image size contributes positively to the model’s ability to generalise and perform well on unseen data.

Examining dropout rates, both ‘0.1’ and ‘0.5’ exhibited similar and optimal outcomes during training. Both configurations achieved a training accuracy of ‘0.99’ and a training loss of ‘0.01’. However, the dropout rate of ‘0.5’ demonstrated superior validation performance, achieving a higher accuracy of ‘0.78’ compared to ‘0.77’ achieved by using a dropout rate of ‘0.1’. Additionally, the ‘0.5’ dropout rate led to a lower validation loss of ‘0.92’, suggesting improved generalisation performance and better prevention of overfitting compared to the ‘0.1’ dropout rate.

In terms of activation functions, the Swish activation function stood out as the most effective. With a training accuracy of ‘0.99’, training loss of ‘0.01’, validation accuracy of ‘0.79’, and validation loss of ‘0.92’, Swish surpassed other activation functions. Its ability to introduce non-linearity to the model while maintaining smoothness contributed to its success.

### 4.3. Optimal Model_STEP 3_

The findings from Section 4.2 provide valuable insights into the impact of different parameters on the DenseNet model’s performance. Specifically, Adagrad as an optimiser, a larger image size of 224×224, a moderate dropout rate of ‘0.5’, and the Swish activation function were identified as optimal choices for deploying the DenseNet model. A comparison of this **optimal** version with the **baseline** is shown in Table 4.

Several notable differences emerged between the **baseline** and **optimal** configurations. The **baseline** setup yielded a training accuracy of ‘0.74’, while the **optimal** configuration demonstrated a substantial improvement, achieving an impressive ‘0.99’. A similar trend was observed in the training loss, where the **baseline** registered ‘1.25’, contrasting sharply with the **optimal**’s significantly lower value of ‘0.01’. Similar trends were replicated in the validation metrics, where the **baseline** accuracy stood at ‘0.68’, while the **optimal** configuration showed a considerable boost to ‘0.79’. Simultaneously, the validation loss in the **baseline** was ‘2.83’, markedly reduced to ‘0.92’ in the optimised version.

**Figure 4:**
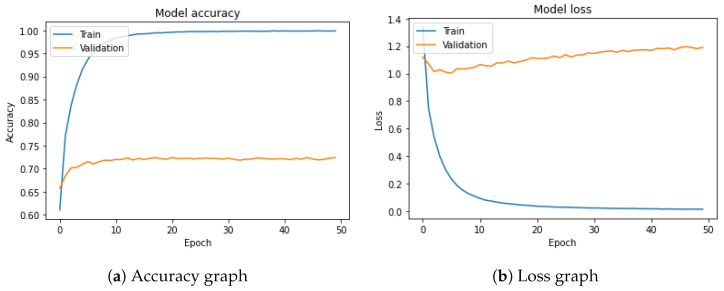
Results of the optimal approach showing accuracy and loss.

Despite these improvements in performance metrics, it is important to note that the runtime also increased in the **optimal** configuration, rising from ‘4150.94’ ms in the **baseline** to ‘6689.80 ms’. This highlights the trade-offs between computational efficiency and enhanced model accuracy. Both experiments were obtained over 50 epochs as shown in Figure 4 but it seems that some of the parameters used in the optimal configuration are complex, leading to higher computational costs.

## 5. Discussion

It is important to put the experiment results into context especially to show how (and to what extent) this study addressed the research aim, which focuses on a multi-class image classification task. Specifically, a discussion is made surrounding the reasons behind the results in Section 4 and its implications for global food waste management.

DenseNet emerged as the preferred architecture based on the **baseline** results in Section 4.1, showing remarkable accuracy (training = 74%, validation = 68%) and computational efficiency (4.1 s). Its dense connectivity pattern facilitates effective feature propagation, leading to accurate learning across diverse food classes. The DenseNet architecture’s success is further evidenced by a reasonable training loss of 1.25 and a validation loss of 2.83, which is indicative of successful convergence without overfitting. In contrast, Xception architecture closely follows DenseNet with a training accuracy of 72% and a validation accuracy of 62%. While Xception leverages depth-wise separable convolutions for efficiency, it comes at a slightly longer runtime of 5097.21 ms, implying a discernible trade-off between accuracy and computational cost. Meanwhile, VGG-16, characterised by its deep architecture, achieves competitive training accuracy (65%) but grapples with challenges in generalisation, ultimately resulting in a lower validation accuracy of 51%. Despite signs of overfitting, VGG-16’s computational intensity is evident in its runtime of 5.2 s.

InceptionNet, which is strategically designed for multi-scale feature capture, achieved a training accuracy of 55% and a validation accuracy of 51%, placing it on par with VGG-16. Notably, InceptionNet achieves this commendable accuracy with enhanced efficiency, boasting a runtime of 2.4 s. This efficiency underscores its proficiency in capturing diverse features across different scales, making it a compelling choice for tasks demanding both accuracy and computational effectiveness. In contrast, MobileNet strikes a harmonious balance between accuracy and efficiency, exhibiting a lower training accuracy of 38% and a validation accuracy of 55%. With a runtime of 2.8 s, MobileNet positions itself as one of the faster models, demonstrating a favourable compromise between computational speed and accuracy. However, VGG-19 encounters challenges similar to VGG-16, grappling with overfitting and a noticeably slower runtime of 7.1 s. This emphasises the inherent trade-off between accuracy and computational cost for these deep learning architectures.

**Figure 5:**
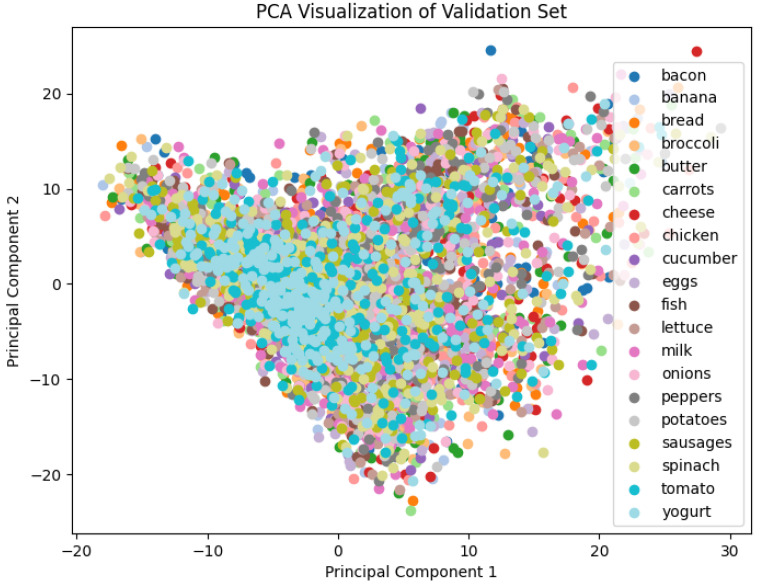
Principal component analysis of the validation dataset.

Surprisingly, ResNet’s performance is notably poor across all metrics, yielding a training accuracy of 9% and a validation accuracy of 11%. This unexpected outcome indicates its inherent difficulties in learning effective residual mappings for the intricate differences of the modest food image dataset as shown in Figure 5. According to Nichani et al. [68], increasing depth in later blocks of ResNet leads to a more drastic increase in test error compared to increasing depth in earlier blocks. This was corroborated by a recent study conducted with ResNet of different layer depths—18, 34, 50, and 152 [69]. The study shows that ResNet performance is determined by the richness of semantic features of the datasets rather than the depth. The study also found that freezing most layers of ResNet and only training the last fully connected layer does not improve the accuracy and efficiency of transfer learning. This emphasises the challenges that ResNet faced in distinguishing between the intricate data composition and proximity between classes. The condensed representation provided by the principal component analysis (PCA) in Figure 5 reveals subtle variations and overlaps, highlighting the need for a detailed understanding of class boundaries. In the context of CNN architectures, these findings highlight the importance of a resilient architecture capable of capturing and learning intricate features to navigate inter-class relationships. Specifically for ResNet, Wang et al. [69] suggested that fine-tuning all layers offers a practical way to reach the best performance based on the amount of available data. This will be explored further in future work but it is worth noting that ResNet exhibits a relatively fast runtime of 4.9 s, highlighting a compromise in accuracy for computational efficiency.

While the unexpected outcome of ResNet prompts a thorough reassessment of its suitability for the particular task examined in this study, this falls outside the comparative analysis aimed at establishing a baseline architecture, where DenseNet emerged as the most accurate and computationally efficient option. The results of parameter tuning on the DenseNet as presented in Table 3, reveal intriguing insights into the impact of various parameters on training and validation performance. In the context of optimisers, the SDG and Adagrad stood out with exceptional training accuracy (99%), outperforming other optimisers. Adagrad, in particular, demonstrated dominance by achieving the best validation accuracy (76%) and the lowest validation loss (0.96), indicating superior generalisation.

The operational characteristics of optimisers play a crucial role in the trajectory of model convergence, particularly in the context of complex multi-class classification tasks. For example, the fixed learning rate of traditional SDG may have hindered its adaptability to varying complexities within different food classes. This reinforces the importance of carefully selecting optimisers based on their operational strategies, with Adagrad proving to be a favourable choice for enhancing the adaptability and generalisation capabilities of DenseNet on the fine-grained food image dataset. The high performance is probably due to Adagrad’s adaptive learning rate approach, which dynamically adjusts learning rates based on historical gradients. Such adaptability has proved pivotal in addressing challenges inherent in multi-class food image classification, where certain features may be infrequent or sporadic across the dataset [70].

Moving onto the impact of image size on performance, it is evident that larger image sizes led to improved accuracy. The transition from 80×80 to 224×224 resulted in a gradual increase in both training and validation accuracy. Notably, the model trained on 224×224 images achieved the best overall performance, with a training accuracy and validation accuracy of 99% and 78%, respectively. This emphasises the importance of image resolution in capturing minute details within images, ultimately enhancing the model’s performance.

Regarding the dropout rate, the model’s sensitivity to dropout values is minimal, as both ‘0.1’ and ‘0.5’ dropout rates yielded comparable high accuracy scores. This suggests that DenseNet is relatively robust to changes in dropout rates for this specific task. However, a dropout rate of 0.5 was chosen as it achieved marginally better performance in terms of validation accuracy and loss.

Exploring activation functions, it is notable that ReLU, Swish, and GELU consistently led in performance across all metrics. These activation functions exhibit strong nonlinear characteristics, aiding in capturing complex relationships within the data. Swish, with its smoothness and non-monotonicity [71], emerged as the optimal choice, achieving the highest validation accuracy (79%) and the lowest validation loss (0.92). This shows the pivotal role of activation functions in shaping the decision boundaries of the model, with Swish standing out as the most effective choice for DenseNet in this food image classification task.

The parameter tuning process, as evidenced in Table 3, guided the selection of Adagrad as the optimal optimiser for DenseNet. The adaptability inherent in Adagrad’s learning rate, particularly its emphasis on infrequent features within the dataset, played a pivotal role in achieving superior generalisation. This adaptability is reflected in the final model’s performance, highlighted in Table 4, where the **optimal** DenseNet configuration yielded substantial improvements compared to the **baseline**. Notably, the **optimal** DenseNet exhibited a training accuracy of 99%, a training loss of 0.01, a validation accuracy of 79%, and a validation loss of 0.92, outperforming the **baseline** in all metrics. The associated runtime of 6.6 s indicates a computational compromise for the enhanced model accuracy. This integration of parameter tuning and subsequent optimisation underscores the crucial role of meticulous parameter selection in overcoming the unique operational challenges posed by multi-class classification tasks, ultimately leading to the realisation of **optimal** model performance.

**Figure 6:**
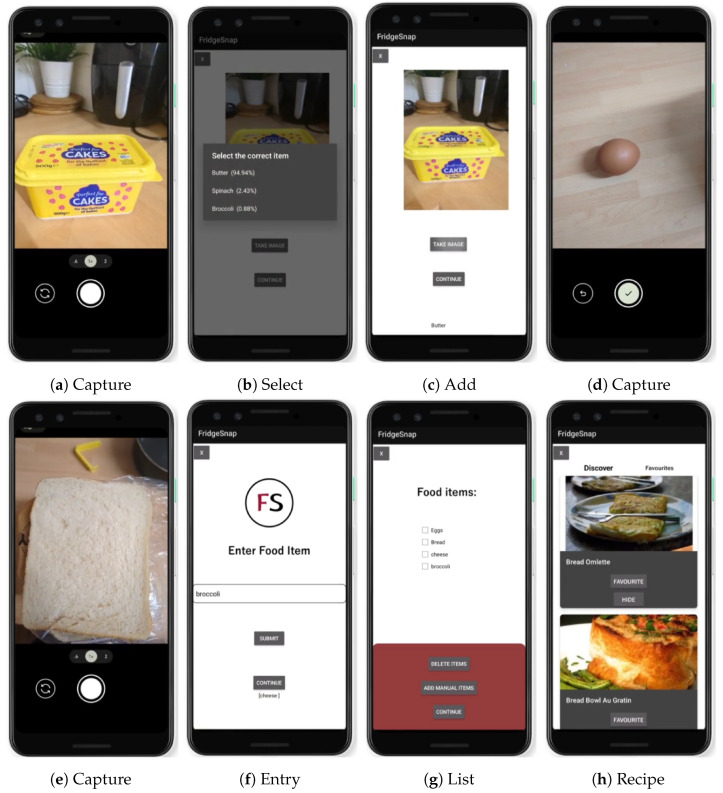
Results of automatic and manual food image capture and recipe suggestion on FridgeSnap.

This study’s achievement in developing an **optimal** DenseNet model represents a significant stride toward practical implementation in real-world applications. Specifically, the **optimal** model, fine-tuned through meticulous parameter selection, now serves as the core image estimator in FridgeSnap [25], a mobile application designed to minimise food waste. This innovative application offers users a seamless experience by allowing them to capture images of food items as shown in Figure 6a,d,e. Each time a food image is captured, the user is asked to select the correct item from a shortlist of predictions presented with confidence levels in descending order (Figure 6b) and the user selection is confirmed (Figure 6c). Each confirmed food is added to an itemised list (Figure 6g) and the user can also enter food items manually as shown in Figure 6f. Leveraging the enhanced capabilities of the **optimal** DenseNet, FridgeSnap provides the user with insightful suggestions for recipes based on the identified contents as shown in Figure 6h. A full demonstration of the application is available on YouTube (FridgeSnap demonstrator available at https://www.youtube.com/watch?v=YhYpCkvr_So (accessed on 29 February 2024)) and the complete integration and deployment source code are available on GitHub [29].

The FridgeSnap application was developed in parallel to the image classification model. The application is further described in [25]. The application works by embedding the developed model in the backend, allowing users to take images of singular food items which are detected and then further processed into recipes using an API. The application was tested without real users using CogTool (https://www.cogtool.org/ (accessed on 29 February 2024)), which allows for replication and evaluation of user actions within the application. Each action is recorded with a timestamp as a measure of success when carrying out usability testing for the application, as users should be able to easily navigate through the application. In this project, common actions within FridgeSnap include the user signing up, signing in, taking an image of an item, and generating a recipe. Table 5 highlights each user action within the application, along with a recorded timestamp.

It is evident from the results that taking an image of an item is more effective for the user, than manually entering the item, with a total difference in time of ‘8.4 s’ between the two actions. The results further emphasise the simplicity of the developed application. Users can sign into the application, take an image of an item, and generate a recipe in less than one minute. However, it is important to note that the time may increase if users have a slower input speed, although this test is not dependent on user input speed.

The deployment of FridgeSnap further serves a purpose beyond user convenience, aligning with a broader commitment to environmental sustainability. By minimising food waste through the thoughtful utilisation of perishables, FridgeSnap contributes to the crucial mission of fostering a more sustainable future. This technological integration, bridging the gap between advanced image recognition and practical utility in everyday life, exemplifies the tangible impact that well-tuned deep learning models can have on addressing pressing societal challenges. It is important to note that the application is not intended to be a comprehensive solution to the global issue of food waste. Rather, it serves as an alternative and a source of support for consumers. It addresses the problem of consumers’ lack of knowledge about food usage by offering a list of easy-to-follow online recipes.

In essence, this study not only advances the understanding of optimal model configurations but also extends its implications into real-world applications, showing the potential of artificial intelligence (AI) in fostering environmentally conscious practices.

## 6. Conclusions

In this study, an exploration is made surrounding the various CNN architectures for the complex task of multi-class classification tailored toward the study’s aim to perform fine-grained food image recognition involving 20 singular food item classes. The approach employed in this task shares similarities with existing approaches (discussed in Section 2) [23,24,37,39], which primarily involves adjustments in pre-trained CNN architectures for improved performance. However, it distinguishes itself by focusing on a more granular classification task. Unlike previous work that often classifies food images into broader categories (such as meat, dairy, and fruit), the task of this study focuses on the classification of individual food items (e.g., carrot, banana, chicken, fish, egg). This poses a unique challenge, given that the complexity of a multi-class classification task increases with the number of classes. In this specific experiment, the intricacies of classifying individual food items (*n* = 20) are tackled, departing from conventional research that typically deals with fewer classes.

The investigation initially explored seven pre-trained CNN architectures that are commonly used in similar studies. However, the attention of this study converged on DenseNet as the architecture of choice due to its exceptional baseline performance on predetermined parameters, including the RMSProp optimiser, ReLU activation function, ‘0.5’ dropout rate, and a 160×160 image size. DenseNet emerged as the front runner, achieving a baseline training accuracy of 74%, validation accuracy of 68%, and computational efficiency of 4.1 s.

Subsequent parameter tuning, encompassing six optimisers, four image sizes, two dropout rates, and five activation functions, further enhanced DenseNet’s capabilities. The **optimised** model demonstrated remarkable improvements, showing a training accuracy of 99%, training loss of 0.01, validation accuracy of 79%, and validation loss of 0.92. The study’s success lies not only in refining insights from exploratory experiments into **optimal** model configurations but also in translating these findings into real-world applications. Specifically, the deployment of the **optimised** DenseNet in FridgeSnap bridges the gap between advanced image recognition and practical utility, exemplifying the tangible impact of well-tuned deep learning models on addressing pressing societal challenges. This technological integration serves as a testament to the potential of AI in fostering environmentally conscious practices and shows the role of CNNs in changing the general approach to food waste management.

## 7. Future Work

Although this study has presented a model that achieved a high accuracy at 79%, it is evident that future work holds promising avenues for exploration and refinement, particularly architectures that exhibited suboptimal performance, such as ResNet, warrant deeper scrutiny. The unexpected underperformance of ResNet across all metrics prompts further investigation into its specific challenges and potential adaptations to better suit the nuances of the fine-grained food image dataset. Future research would involve the development of a custom CNN architecture or the application of current methods in a novel context, for example through fine-tuning ResNet’s structural parameters or considering ensemble approaches to harness the strengths of multiple models.

Additionally, expanding the experimental dataset to encompass a more extensive array of singular food items holds the potential to enhance the model’s ability to generalise across diverse culinary items. A broader range of food items in the dataset could introduce new challenges and intricacies, offering a more comprehensive evaluation of the selected CNN architectures. This expansion could involve incorporating regional or cultural variations in food items, capturing a richer diversity that aligns more closely with real-world scenarios. Moreover, increasing the dataset size may contribute to mitigating overfitting concerns and improving the models’ performances. In fact, an effective way of improving the dataset variation is through image augmentation [56]; this will be explored in future work.

## Figures and Tables

**Table 1:** Dataset composition with class distribution and split.

Food item class	Total	Training	Testing	Validation
Bacon	2040	1306	326	408
Banana	1929	1236	308	385
Bread	2037	1304	326	407
Broccoli	2063	1321	330	412
Butter	2115	1353	339	423
Carrots	2486	1592	397	497
Cheese	2052	1314	328	410
Chicken	2042	1308	326	408
Cucumber	1965	1258	314	393
Eggs	2205	1412	352	441
Fish	2016	1291	322	403
Lettuce	2064	1322	330	412
Milk	2295	1629	407	259
Onions	2224	1424	356	444
Peppers	2022	1295	323	404
Potatoes	2267	1452	362	453
Sausages	2052	1314	328	410
Spinach	2016	1291	322	403
Tomato	2028	1299	324	405
Yogurt	2031	1300	325	406
Total	41,949	27,021	6745	7779

**Table 2:** Results of the comparative analysis between seven pre-trained CNN architectures.

CNN	Training Accuracy	Training Loss	Validation Accuracy	Validation Loss	Runtime (ms)
DenseNet-(**baseline**)	**0.74**	**1.25**	**0.68**	**2.83**	**4150.94**
Xception	0.72	1.25	0.62	3.33	5097.21
VGG-16	0.65	1.55	0.51	8.47	5211.48
InceptionNet	0.55	1.79	0.51	3.05	2495.73
MobileNet	0.38	2.49	0.55	2.34	2859.40
VGG-19	0.53	1.93	0.45	6.74	7187.57
ResNet	0.09	2.94	0.11	11.99	4919.54

Note: Accuracy results are represented as floating point values between 0 and 1. The best result on each metric is highlighted in **bold** type font. This is the **baseline** also highlighted in ‘grey’. Experiments were conducted with batch size: ‘16 ’, epoch: ‘50’, image size:‘160×160’, optimiser: ‘RMSProp’, activation function: ‘ReLU’, dropout: ‘0.5’ and learning rate: ‘0.001’.

**Table 3:** Results of parameter tuning on DenseNet CNN architecture.

Parameter		Training Accuracy	Training Loss	Validation Accuracy	Validation Loss	Runtime (ms)
OPTIMISER	**SDG**	0.99	0.02	0.76	1.63	3711.01
	**Adam**	0.77	0.69	0.70	2.43	3823.47
	**RMSProp**	0.74	1.25	0.68	2.83	4150.94
	**Adagrad**	**0.99**	**0.04**	**0.76**	**0.96**	**3811.85**
	**Adamax**	0.99	0.04	0.75	1.87	3893.97
	**Nadam**	0.78	0.66	0.70	2.35	4757.08
IMG_SIZE	**80 × 80**	0.81	0.63	0.65	1.21	1331.29
	**120 × 120**	0.95	0.21	0.72	1.03	2149.88
	**160 × 160**	0.99	0.04	0.76	0.96	3811.85
	**224 × 224**	**0.99**	**0.01**	**0.78**	**0.92**	**6644.99**
D_OUT	**0.1**	0.99	0.01	0.77	1.02	6615.02
	**0.5**	**0.99**	**0.01**	**0.78**	**0.92**	**6644.99**
ACTIVATION	**ReLU**	0.99	0.01	0.78	0.92	6644.99
	**Sigmoid**	0.98	0.12	0.77	0.83	6651.85
	**Tanh**	0.99	0.02	0.77	0.98	6735.93
	**Swish**	**0.99**	**0.01**	**0.79**	**0.92**	**6689.80**
	**GELU**	0.99	0.01	0.78	0.88	6670.22

Note: All results are represented as floating point values between 0 and 1 except for ‘Runtime’. The best result on each parameter is highlighted in **bold** type font.

**Table 4:** Baseline vs. final results.

CNN	Training Accuracy	Training Loss	Validation Accuracy	Validation Loss	Runtime (ms)
DenseNet (baseline)	0.74	1.25	0.68	2.83	4150.94
DenseNet (optimal)	0.99	0.01	0.79	0.92	6689.80

Note: Accuracy results are represented as floating point value between 0 & 1. The best overall result highlighted in ‘grey’ was produced by the **optimal** model, albeit with higher runtime than the **baseline** model.

**Table 5:** CogTool results.

Task	Average Time Taken to Carry out Task
Sign up	4.7 s
Reset password	7.1 s
Sign in	1.7 s
Take image of item	5.4 s
Add item manually	13.8 s
Remove item from list	13.6 s
Search for recipes	13.6 s
Open favourite recipes	15.4 s
Open discovered recipes from favourites	16.9 s

## Data Availability

The original data used for this research are freely available on the Kaggle dataset repository, entitled the multi-class food image dataset [25]. To encourage reproducibility of the experiments and results reported in Section 4, the source code used for the experiments was deposited at [29].
